# Astrocyte epigenetics in development, aging, and neurodegeneration: a DNA methylation perspective

**DOI:** 10.3389/fnmol.2026.1876219

**Published:** 2026-07-10

**Authors:** Uchit Bhaskar, Melanie A. Carless

**Affiliations:** 1 Department of Neuroscience, Developmental and Regenerative Biology, The University of Texas at San Antonio, San Antonio, TX, United States; 2 Brain Health Consortium, The University of Texas at San Antonio, San Antonio, TX, United States

**Keywords:** aging, astrocyte, DNA methylation, epigenetics, neurodegeneration, neurodevelopment

## Abstract

Epigenetic modifications, including DNA methylation, have long been associated with developmental programming, as well as aging and disease states. However, our understanding of cell-specific epigenomic landscapes remains limited, especially in the context of brain aging and neurodegeneration. In cases of late-onset brain disorders, such as Alzheimer’s disease, progressive supranuclear palsy, Parkinson’s disease, and frontotemporal dementia, unraveling cell-specific epigenomic contributions is particularly necessary to better understand the molecular contributors to early disease states, which may help enhance diagnostic and therapeutic measures. While whole brain tissue and neuronal cell-type-specific methylomic contributions have been extensively studied, those of glia, including astrocytes, remain poorly elucidated. Given the key role of DNA methylation in guiding neurodevelopmental timing and gliogenic onset, it is likely that these modifications alter astrocyte functionality with age and disease. Here, we briefly review astrocyte development in the context of DNA methylation and highlight key instances where methylomic changes contribute to astrocyte maturation and functionality. We also point to evidence showing extensive transcriptomic and functional changes associated with aged and diseased astrocytes and explore the relevance of DNA methylation in these conditions. Ultimately, elucidating molecular drivers of disease states in astrocytes will allow for a better understanding of cell-specific contributions and pave the way for future research directed at cell-specific therapeutics.

## General overview

Emerging evidence suggests that understanding overall brain health requires a longitudinal perspective. This is primarily because neurodevelopmental programming and adult brain aging appear to be intrinsically linked mechanisms that drive nervous system function and, consequently, neurodegeneration-like pathological states. Such disorders are primarily characterized by progressive loss of cell function that leads to nervous system dysfunction, and encompass a wide variety of disorders, including Alzheimer’s disease (AD), frontotemporal dementia (FTD), Parkinson’s disease (PD), progressive supranuclear palsy (PSP), and others. Since neurodegenerative disorders are primarily a result of neuronal loss, these cells have been the central focus of most studies and the role and involvement of glial cells are less well studied. Astrocytes, one type of glial cell, are well-known central players in brain homeostasis, involved in the maintenance of the blood-brain barrier, regulation of synapse formation and neurotransmission, and control of metabolic and ionic fluxes. With age and under pathological conditions, astrocytes become atrophic and exhibit reactive states, contributing to impaired synaptic transmission and neuronal plasticity, as well as a loss of central nervous system (CNS) homeostasis, which further drives neuroinflammation and neurodegeneration. However, the mechanisms by which astrocyte dysfunction contributes to neurodegeneration are not well elucidated.

Epigenetic modifications are consistently associated with different neuropathological conditions, including neurodegenerative disorders. Epigenetic mechanisms, such as DNA methylation and histone modifications, allow the regulation of gene expression without modifying the underlying genetic sequence. DNA methylation, the focus of this review, refers to the addition of a methyl group on a deoxyribose-cytosine, most commonly within a cytosine-guanine (CpG) dinucleotide context. In general, CpG methylation at promoter and enhancer regions of genes tends to downregulate gene expression, whereas those at other locations in the genome may be responsible for processes such as alternative splicing and transposable element silencing (Jones, 2012). Although DNA methylation at CpG-dense promoters can enforce stable transcriptional repression, this relationship is nuanced and highly dependent on the genomic context, CpG density, chromatin state, transcription factor occupancy, and cell type (Wagner et al., 2014; Moarii et al., 2015; Heery and Schaefer, 2025). Methylation at CpG sites occurs through the action of DNA methyltransferases (DNMTs), which transfer a methyl group from an S-adenosyl methionine residue onto the fifth carbon atom of a cytosine at CpG sites. Conversely, active DNA demethylation is mediated by the enzymatic activity of the ten-eleven translocase (TET) family of proteins. While DNA methylation patterning is critical during development for cell- and tissue-type specification, changes in DNA methylation signatures have also been associated with disease states. Studies have observed accelerated epigenetic aging in neurodegenerative disorders, and epigenome-wide association studies (EWAS) have elucidated a key role for DNA methylation in both aging and neurodegeneration.

Recent research in the field of aging and neurodegeneration has begun to implicate glia-specific epigenetic alterations. These studies are still in their infancy, and further exploration in this area is required to fully comprehend the extent to which cell-specific contributions alter disease processes. Here, we discuss how DNA methylation impacts astrocyte development and function, and how these modifications may contribute to aging and neurodegeneration. Throughout this review, we consider astrocyte DNA methylation as a regulatory process that operates along a continuum involving cell fate establishment, maintenance, and potential destabilization with aging and neurodegenerative disease. We detail how, during CNS development, methylation and demethylation events regulate the timing of gliogenic competence, as well as astrocyte maturation and functionality. We also highlight how, with aging and neurodegeneration, these regulatory systems may be remodeled through cell-intrinsic epigenetic drift, altered metabolic states, inflammatory signaling, or via reactivation and suppression of developmental pathways. Ultimately, we propose that decoding the astrocyte methylome represents a critical frontier for uncovering potential drivers of aging and neurodegeneration, establishing pathogenic mechanisms, and identifying novel therapeutic strategies.

## Astrocytes in CNS development

**FIGURE 1:**
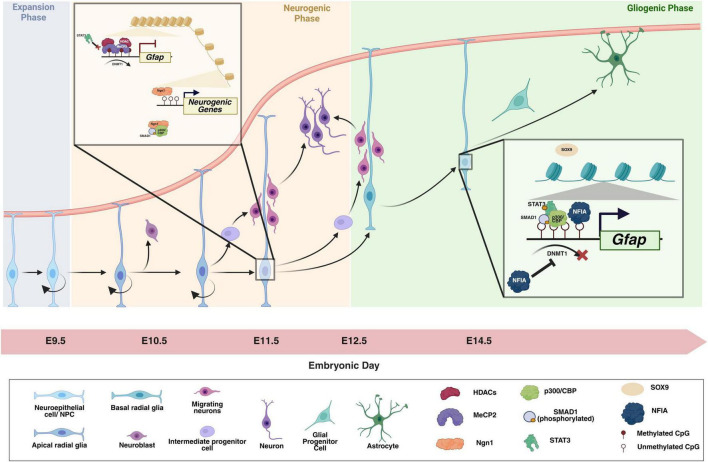
Schematic of DNA methylation contributions to astrocyte development. Steps involved in neurogenesis and gliogenesis are shown. Within the neurogenic window, radial glial cells exhibit DNA methylation at promoter regions of astrocyte-related genes (*e.g., Gfap*), mediated by DNMT1. This causes binding of MeCP2 and histone modifiers, that prevent the localization of STAT-binding proteins, and therefore prevent glial differentiation. At the same time, neurogenic transcription factors (*e.g., Ngn1*) bind to the SMAD1-p300-CBP complex, preventing recruitment of STAT proteins, and promoting neurogenesis. During late stages of embryonic development, SOX9 expression promotes changes to chromatin conformation, and expression of NFIA inhibits the activity of DNMT1, thereby, removing methylation marks on the *Gfap* promoter. This allows binding of STAT3-SMAD-p300/CBP complexes, as well as NFIA, driving glial lineage transcription. This figure was created using Biorender.

Originally thought to be passive support cells, serving as a “neural glue” (Somjen, 1988), astrocytes are now recognized as a heterogeneous population of cells, unified by their role in maintaining CNS homeostasis (Allen and Barres, 2009). Arising from a common neuroepithelium, they are thought to comprise nearly 20–40% of all cell types in the adult brain (von Bartheld et al., 2016). The developmental trajectory of astrocytes is a complex, interwoven process involving cell-intrinsic epigenetic modifications and transcription factor expression, as well as cell-extrinsic signaling cues that enable fate specification. Below, we provide an overview of the astrocyte development trajectory and highlight key instances where DNA methylation plays a critical role in defining astrocytic cell fate (Figure 1).

### A role for transcription factors in astrocyte fate specification

During mammalian nervous system development, neuroepithelial cells arise from a neural tube, generating a lineage-restricted, multipotent progenitor population termed neural progenitor cells (NPCs). NPCs undergo a distinct temporal fate specification during development, accompanied by dramatic DNA methylation and hydroxymethylation changes (for a detailed review on hydroxymethylation, see Bhaskar et al., 2025), where cellular differentiation in the mid-gestational window is exclusively neurogenic, but during late-gestation and postnatally, gives rise to astrocytes and oligodendrocytes. These distinct phases are supported by changes in the epigenomic landscape, as well as transcription factor-relevant cues and other extrinsic signals (reviewed in detail in Sloan and Barres, 2014). Broadly speaking, studies focused on the ventricular zone of the murine spinal cord suggest a cessation of neurogenesis around embryonic day E11.5, and a gliogenic commitment starting around E12.5, which coincides with the activation of SRY-box transcription factor-9 (*Sox9*) and nuclear factor-1 alpha (*Nfia*) (Kang et al., 2012). Activation of Notch signaling in neural stem cells creates a permissive environment for astrocytogenesis and promotes *Nfia* expression, which, together with *Sox9*, regulates a complex transcriptional cascade promoting astrocyte development (Deneen et al., 2006; Kang et al., 2012). In the neocortex, studies also point to a role for bHLH transcription factor, *Olig2*, in both astrocyte and oligodendrocyte fate-specification (Marshall et al., 2005; Ono et al., 2008), where co-expression of *Olig2* and *Sox9*, along with a BTB/POZ family transcription factor, *Zbtb20*, directs astrocyte fate choice over oligodendrocytes (Nagao et al., 2016). Importantly, the sole expression of these transcription factors is insufficient for astrocyte differentiation, and extrinsic cell signaling cues from the JAK-STAT and BMP pathways, as well as the regulation of epigenetic states in the developing NPCs and astrocyte progenitors, appear to be critical. For example, chromatin conformation favorable to gliogenic induction, mediated by a *Sox9/Brn2* regulatory effect, appears to precede *Nfia* expression, at least in the ventricular zone (Glasgow et al., 2017), which suggests that concerted epigenomic-transcriptomic efforts are necessary for both gliogenic competence and differentiation.

### DNA (de)methylation is key to gliogenic competence during neurodevelopment

Early studies in the field show that Notch-mediated activation of Nfia promotes demethylation of astrocyte-relevant gene promoters and enhancers (including Gfap) in NPCs (Namihira et al., 2009). A follow-up study reinforced this idea by showing that the NFI expression in NPCs confers a genome-wide demethylation state that enhances their gliogenic competence (Sanosaka et al., 2017), and experimental evidence from human induced pluripotent stem cell (iPSC)-derived NPCs also shows that a transient induction of NFIA in NPCs is sufficient to drive the gliogenic program (Tchieu et al., 2019). However, to induce differentiation from this permissive state, signaling cues from the IL6-family of cytokines (e.g., CNTF, LIF, and CT-1), which activate either STAT1 or STAT3 proteins, are required (Barnabé-Heider et al., 2005; He et al., 2005). This, along with BMP signaling during late embryonic and postnatal windows, promotes astrocyte differentiation. BMPs, through downstream SMAD activation, synergize with STAT3 and activate the GFAP promoter via a STAT3-p300-SMAD1 complex (Nakashima et al., 1999). Further, BMP-induced expression of the HES and ID family of proteins limits oligodendrocyte lineage development through sequestration of OLIG1/2 within the cytoplasm, and inhibition of neuronal differentiation by downregulation of other bHLH family transcription factors, including Mash1, NeuroG1, and NeuroD (Gross et al., 1996; Mehler et al., 2000; Nakashima et al., 2001; Bond et al., 2012).

These processes are largely driven by dynamic epigenetic changes occurring in neuroepithelial cells during development. At early stages of neurogenesis in mice (∼E11.5), CpG sites in the promoters of genes such as *Gfap* are methylated by a maintenance DNA methyltransferase (DNMT1). This prevents binding of STAT3 activator complexes, which are required to drive expression of astroglia-specific gene programs. Curiously, even in the presence of STAT proteins at early stages of neurogenesis, *Gfap* gene expression is non-existent due to this epigenetic regulation. Seminal studies in the astrocyte development field show that the loss of CpG methylation at these sites is a required and necessary step for the functionality of STAT-mediated signaling (Takizawa et al., 2001; Fan et al., 2005; Hatada et al., 2008). This is supported by the fact that during early time points of neurodevelopment (∼E11.5), the methylated STAT binding sites on promoters of astroglial lineage genes appear to be tightly associated with the methyl-CpG binding protein (MeCP2), which is known to recruit histone modifiers to confer an inactive chromatin state, thus preventing gene expression (Fan et al., 2005). Late-stage NPCs (∼E14.5) exhibit a demethylated state at astrocyte gene promoters that lack a MeCP2 binding program, therefore creating a permissive environment and allowing subsequent STAT3 binding to drive differentiation of the gliogenic lineage (Hatada et al., 2008). This is also observed for other astrocyte-relevant genes, including *S100B* (Namihira et al., 2004). Consistent with this, experimental evidence suggests that ascorbic acid-induced DNA demethylation, or the inhibition of DNA methyltransferases and histone acetylases in NPC cultures, can enhance glial differentiation (Majumder et al., 2013; Kim et al., 2018). This is, at least in part, due to the formation of hydroxymethyl cytosine intermediates at consensus binding motifs of *NFIA*, which significantly enhances recruitment of NFI and STAT3 to the promoter sites of glial lineage genes (Kim et al., 2018). Others also show that exposing human iPSC-derived NPCs to a hypoxic environment promotes cooperation between HIF1α and NFIA, accelerating DNA demethylation and promoting gliogenesis (Yasui et al., 2017).

It is interesting to note that astrocytes express low levels of *de novo* DNA methyltransferases (e.g., DNMT3A) compared to other brain cell types, with the loss of DNMT3A in NPCs promoting gliogenesis (Feng et al., 2005; He et al., 2020). This suggests that a lack of DNA methylation in NPCs is critical in the fate-switch from neurogenesis to gliogenesis. Crucially, recent studies have also highlighted that Tet-dependent active DNA demethylation during neurodevelopment may be critical for poising gliogenic enhancer programs, and a failure to do so compromises NPC identity (Wheldon et al., 2014; MacArthur et al., 2024). Studies also show that CpG sites at glial gene promoters, including those at *Gfap* and *Olig2*, acquire carboxyl and formyl cytosine states (both demethylation intermediates) during differentiation (Murao et al., 2016). It remains unclear whether the acquisition of gliogenic competence in NPCs through TET-mediated demethylation is a prerequisite for NFI-mediated activation of transcriptional programs, given that NFIA binding at astrocytic gene promoters has been reported to promote demethylation by dissociating DNMT1 (Namihira et al., 2009). It is possible that both active DNA demethylation mediated by TET enzymes and the reduction in methylation facilitated by NFI-mediated blocking of DNMT1 binding sites might occur concurrently, and that both processes play a role in gliogenic cell fate specification. Furthermore, other epigenetic modifications, including histone and chromatin modifications and small non-coding RNAs (see Neal and Richardson, 2018; Yoon et al., 2018 for a detailed review), also play critical roles in neurodevelopmental processes, and ultimately, the combined effects of all these epigenomic cascades underlie the cell fate determination process.

### DNA methylation in astrocyte maturation and functionality

Following gliogenic fate induction in NPCs, the activity of JAK/STAT signaling molecules, as well as BMPs, promotes differentiation to astrocyte precursor cells and mature astrocytes. Fetal development is characterized by a proliferative astrocyte precursor cell state (Ge et al., 2012; Tien et al., 2012; Zhang et al., 2016), which eventually matures during postnatal stages to a non-proliferative state, coincident with the expression of mature astroglial markers. It is also important to note that after maturation to astrocytes, genes and transcription factors critical to early gliogenic fate specification (e.g., *Dll1*, *Hes1*, *Hes5*) seem to be hypermethylated, further strengthening gliogenic commitment (Hatada et al., 2008). How this hypermethylation occurs, given the low levels of *de novo* DNMTs in mature astrocytes, is unclear, and further studies are required to elucidate such mechanisms. A recent study also showed that while the mature astrocyte cell fate is “locked-in” after differentiation, in cases of ischemic brain injury and other insults, these fate-locking mechanisms can be released to generate neuroblasts (Kremer et al., 2024), suggesting that the methylation states may be amenable to modulation even in mature cell types. Ultimately, these instances point to a critical role for DNA methylation in not just the initiation of gliogenesis and the differentiation and maturation to astrocytes, but also in maintaining a fate-specified cell state.

Mature astrocytes in the postnatal brain express genes involved in glutamate uptake (*GLT1, SLC1A2, SLC1A3, GLUL, GS*), gap junction communication (*GJA1, Cx43, Cx30, GJB6*), water and ionic homeostasis (*AQP4, KIR4.1)* and synapse formation and maturation (*SPARCL1* and Glypicans), as well as those that act as transmembrane receptors (e.g., *FGFR3, GABRA2*) or encode secretory proteins (*THBS1, CLU, SPON1*, and GPCs) (Yamamoto et al., 1992; Furuta et al., 1997; Nagy et al., 1999; Seifert et al., 2009; Clarke and Barres, 2013; Fallier-Becker et al., 2014; Zhang et al., 2016; Farhy-Tselnicker and Allen, 2018; Akdemir et al., 2020). Largely, astrocyte functionality in a healthy adult brain is highly dependent on their regional localization and microenvironment. Essentially, astrocytes in proximity to neurites and synapses are involved in neurotransmitter and ionic buffering; those proximal to other astrocytes engage in metabolite and signaling exchange, contributing to synchronized homeostatic responses; those proximal to microglia promote axon-guidance and cytokine responses; and astrocyte end-feet at the blood-brain-barrier regulate neurovascular coupling and blood-brain-barrier integrity (Kwon et al., 2025).

It is not surprising that DNA methylation at the promoters of some astrocyte-specific genes (e.g., *Slc1a2, Glul, Lcat*) is low or absent in mature states, promoting gene expression and subsequent functionality, as evidenced by recent studies in rodent astrocytes of the cortical and sub-ventricular zones, as well as in human glioma cell lines (Zschocke et al., 2007; Kremer et al., 2024). There is also evidence suggesting a role for methylation-dependent regulation of gene expression for the astrocytic potassium channel protein, Kir4.1 (encoded by *Kcnj10*) (Nwaobi and Olsen, 2015). DNA methylation contributes to neuroinflammatory processes, especially in pathological states, where cytokine activation causes methylation-mediated repression of NRF2 signaling to sustain a pro-inflammatory state that is associated with a loss of homeostatic response (Linnerbauer et al., 2020; Wheeler et al., 2020; Gómez Cuautle et al., 2026). In fact, in the healthy brain, decreased methylation is also observed at the promoters of homeostatic response genes, including those involved in lactate shuttle and glutamate processing (e.g., *Ldha, Slc16a1, Gs*), as well as water and ionic homeostasis (e.g., *Aqp4*); these sites seem to be hypermethylated in pathological states, as well as in neuropsychiatric conditions, suggesting the existence of dynamic epigenetic control in overall astrocytic functionality (Nagy et al., 2015; Cuautle et al., 2024). Deciphering these baseline developmental methylation programs is also crucial in our understanding of glioma-like pathological states, where aberrant hijacking of these exact pathways frequently characterizes abnormal, mitotic glial proliferations. Nevertheless, it is important to note that, in addition to methylation, other studies have also shown involvement of chromatin-based transcriptional organization in astrocyte maturation (Lattke et al., 2021). Overall, this suggests that epigenetic modifications are key players in maintaining functional states of mature cell types.

Single-cell and spatial transcriptomic studies show that astrocytes comprise several molecularly distinct regional and intra-regional subtypes that are linked to specialized morphology, synaptic interactions, calcium signaling, neurovascular positioning, and disease susceptibility (Batiuk et al., 2020; Habib et al., 2020; Brandebura et al., 2023; Kwon et al., 2025). Because DNA methylation is a stable marker of both lineage history and cellular state, methylomic profiles from astrocytes are likely to capture a mixture of developmental origin, regional specialization, maturation state, and disease-associated reprogramming, demonstrating that astrocyte heterogeneity is central to interpreting astrocyte methylomes. Consequently, future studies should avoid treating astrocytes as a single uniform group and integrate region, localization, pathology, sex, age, and reactive-state markers whenever possible.

## A role for astrocytes in aging

The developmental mechanisms described above provide a useful framework for understanding aging in astrocytes. Historically, astrocytes have been known to mount a rapid, reactive response in the CNS to acute insults (e.g., viral infections, traumatic injury), age-related cognitive decline, and neurodegenerative conditions and associated pathologies (e.g., amyloid in AD, tau in PSP). Such reactive states are marked by increased GFAP levels and dramatic morphological changes, ultimately limiting the ability of cells to maintain a homeostatic environment in the brain. Often, these are accompanied by changes in the epigenetic architecture of the cells. During gliogenesis, DNA methylation helps regulate when astrocyte lineage genes become accessible and how mature astrocyte identity is established. With aging, astrocytes do not simply lose function uniformly but rather undergo cell-state changes that include reduced homeostatic and metabolic support, altered regional identity, inflammatory signaling, and reactive remodeling. These age-associated phenotypes may involve partial disruption of the epigenomic programs that maintain astrocyte identity, as well as engagement of developmental or injury-associated pathways. In this section, we will briefly explore astrocytic dysfunction during aging and how DNA methylation is relevant to such age-related processes.

### Age-related dysfunction in astrocytes

Recent studies of the aging mouse brain, as well as the human brain, show that age-associated changes in the CNS largely stem from glial cell types, including astrocytes and oligodendrocytes (Soreq et al., 2017; Allen et al., 2023; Hahn et al., 2023). Astrocytes typically enter an A1-like reactive state with aging, concomitant with increased expression of general reactive state markers (e.g., *GFAP, Serpin3n*), and other inflammatory proteins (e.g., C3) (Boisvert et al., 2018; Clarke et al., 2018). Some of these changes are accompanied by morphological and functional alterations, including defects in glutamate and potassium ion clearance, and calcium signaling in aged astrocytes (Popov et al., 2021). Several studies also point to region-specific changes in astrocytic gene and protein expression during aging (Boisvert et al., 2018; Clarke et al., 2018; Ximerakis et al., 2019). For example, studies show that hippocampal and striatal astrocytes in the mouse brain have more differentially expressed genes with age compared to cortical astrocytes (Clarke et al., 2018), and astrocytes in the cerebellum and hypothalamus have more age-related expression changes than those in the cortex (Boisvert et al., 2018). Boisvert and colleagues show consistent downregulation of genes relevant to the cholesterol biosynthesis pathway in astrocytes with age, and upregulation of genes involved in synapse elimination processes, suggesting the existence of an environment that limits normal synaptic functionality. In support of this, studies using primate models, as well as those profiling the human dorsolateral prefrontal cortex, also show reduced expression of genes involved in synaptic modulation and neuronal support (Chiou et al., 2022; Ling et al., 2024). Immunological pathways exhibit substantial changes in aged astrocytes, at least in rodent models (Allen et al., 2023). Such changes seem to, in part, occur as a response to a microglial inflammatory phenotype (Clarke et al., 2018). Overall, homeostatic astrocyte functions are hampered with aging and age-related processes, decreasing optimal maintenance of neuronal support and metabolism, which is likely accentuated in disease processes (see next section). However, the unique role of epigenetic modifiers in aging is less well understood in a cell-type-specific context. Below, we highlight recent studies exploring DNA methylation changes in glial cell types, occurring with age. Detailed reviews of the overall effects of aging on astrocytes can be found elsewhere (Rodríguez-Arellano et al., 2016; Palmer and Ousman, 2018; Labarta-Bajo and Allen, 2025).

### Age-associated DNA methylation changes in glial cells

Age-associated changes in DNA methylation are well documented across multiple tissue types (Horvath, 2013). Notably, CpG sites that show consistent methylation differences with age, and across tissues, collectively termed “epigenetic clocks”, have been used extensively as predictors of chronological and biological age (Hannum et al., 2013; Horvath, 2013; Levine et al., 2018; Lu et al., 2019). In the human cerebral cortex, studies have shown a dynamic regulation of DNA methylation across the lifespan, at least in neurons (Siegmund et al., 2007; Chien et al., 2024). Other studies have shown robust region-specific differential DNA methylation in aging brains (Pellegrini et al., 2021). One study, focusing on EWAS in aging and AD, separated cell fractions into neuronal (NeuN+) and non-neuronal (NeuN-) subtypes and observed interesting age-associated DNA methylation changes. For instance, only a small fraction of age-related differentially methylated sites are common between neuronal and non-neuronal fractions (i.e., 49 of the top 1000 CpG sites), and these often depict opposing methylation trends. Non-neuronal cells showed differential DNA methylation in age-associated genes such as *ELOVL2, FHL2, RAI1, CUX1*, and others, but these were not observed for neurons, suggesting the existence of age-associated cell-type-specific methylation dynamics (Gasparoni et al., 2018). In fact, in a recent deconvolution-based EWAS meta-analysis (non-peer-reviewed preprint), our group observed a significantly large proportion of hypermethylated CpGs associated with age in prefrontal cortex astrocytes compared to other cell types; several of these map to known homeostatic astrocyte function genes, including *ATP1A1, CACNA1C, FBLN5*, and others (Bhaskar et al., 2026). Other studies show chromatin remodeling and altered histone dynamics associated with age in astrocytes, suggesting potential large-scale epigenome-wide modifications in these cell types (Chisholm et al., 2015; Neal and Richardson, 2018; Palmer and Ousman, 2018; Morales Pantoja and Mintz, 2026). These findings underscore the necessity of investigating age-associated DNA methylation profiles within specific cell populations.

Given that astrocytes are a heterogeneous population that varies across brain regions, such region-specific findings have important implications for astrocyte methylation studies. Methylation signatures measured from a given brain region may reflect local developmental history, neuronal activity, vascular niche, inflammatory exposure, and the relative abundance of distinct astrocyte states. Thus, age-associated methylation changes in astrocytes may differ across different brain regions, paralleling the region-specific transcriptional and functional changes observed with age. This also raises the possibility that some methylation changes reflect broad astrocyte programs, whereas others may represent regionally restricted remodeling of astrocyte identity or function.

Taken together, these studies suggest that aging is accompanied by methylomic remodeling in glial cell populations, but care is needed in interpreting these findings. To date, direct evidence for age-associated DNA methylation changes in purified astrocytes remains scarce, with most data being derived from bulk tissue, NeuN- cell fractions, or computational deconvolution methods. Although these studies can identify robust age or disease-associated CpGs, they cannot determine whether the observed differences in DNA methylation are astrocyte-intrinsic or reflect altered astrocyte abundance and/or shifts in astrocyte sub-states. This concern extends to epigenetic clocks, which have been estimated in bulk tissue that is affected by shifts in cell composition and cell states in response to aging and disease (Shireby et al., 2020; Murthy et al., 2023b; Zhang et al., 2023; Tong et al., 2024). While deconvolution methods and newer methylation atlas approaches can partially address this issue, studies of sorted-cell or single-cell/single-nucleus methylome, multiomic, and spatial data remain necessary to distinguish cell-intrinsic aging from cell-population or cell-state remodeling. Nevertheless, several observations across these methods converge on a model where age-related DNA methylation changes in astrocytes may influence homeostatic programs, including ion transport, calcium signaling, and metabolic support. These overlap with transcriptional programs known to decline with aging, suggesting that DNA methylation may participate in maintaining or destabilizing mature astrocyte functions. Future studies that jointly profile methylation, chromatin accessibility, and gene expression in purified astrocytes will be necessary to determine whether these methylation changes are causal drivers, downstream consequences, or stable markers of age-associated astrocyte remodeling.

One striking limitation in studying age-associated phenotypes relevant to the human brain is the lack of suitable model systems. Often, access to human post-mortem tissue is limited, and variability in post-processing curtails its utility. And while deconvolution-based approaches offer insights into likely methylomic changes, these require rigorous validation in a biological system. iPSC-based systems, both 2D cell cultures and 3D organoids, while offering a suitable *in vitro* platform to model different cell and tissue types, tend to lack age-related patterns and are thought to mimic embryonic cell types. In light of this, over the past decade, some groups have generated induced neurons (iNs) and other somatic cell types by direct lineage conversion from human somatic cells, like fibroblasts (Koch, 2015; Mertens et al., 2015; Herdy et al., 2019). Epigenetic clock-based metrics suggest that iNs retain age-related methylation patterns that are lacking in iPSC-derived neurons from the same individuals (Mertens et al., 2015, 2018, 2021), suggesting that directed differentiation might serve as a potential tool for modeling age. Similar models for astrocytes are limited and lack methylomic validation (Caiazzo et al., 2015; Tian et al., 2016; Quist et al., 2022). Future studies addressing these limitations might allow for assessing age-related DNA methylation dynamics in astrocytes and other brain cell types.

## Involvement of astrocytes in neurodegenerative diseases

Given that aging is a major risk factor for the development of neurodegenerative disorders, it is not surprising that glial cells exhibit disease-relevant phenotypes with age. Broadly, aged astrocytes exhibit degenerating, atrophic, and reactive properties that fail to support a healthy CNS. These changes alter physiological astrocytic functioning and hamper CNS homeostasis, resulting in a disease-promoting environment. In the following section, we will first briefly discuss the role of astrocytes in neurodegenerative disorders, with a focus on their pathological state, and shed some light on DNA methylation-related changes that accompany these processes.

### Neuropathological and functional changes in astrocytes during neurodegeneration.

Neurodegenerative disorders are characterized by abnormal accumulation of misfolded proteins (e.g., Aβ and pTau in AD, *α-*Synuclein in PD, TDP-43 in FTD), which disrupt cellular functions and trigger neurodegeneration. Disease-associated astrocytes, in particular, show impairments in glutamate uptake, vesicular transport mechanisms, and synapse maintenance (Díaz-Amarilla et al., 2011; Stenovec et al., 2011) in amyotrophic lateral sclerosis (ALS); impaired calcium signaling and increased apoptosis in FTD (Su et al., 2000; Velebit et al., 2020); increased reactivity and mitochondrial abnormalities in PD (Gelders et al., 2018; Bantle et al., 2020); and atrophy and loss of potassium ion buffering and glutamate uptake in Huntington’s disease (HD) (Hodges et al., 2006; Osipovitch et al., 2019). In AD, astrocytes have been shown to acquire reactive and atrophic states in response to amyloid accumulation, triggering an inflammatory response that contributes to impaired synaptic transmission and neuronal plasticity (Hu et al., 1998; Rodríguez-Arellano et al., 2016; Verkhratsky et al., 2019; Park and Barrett, 2020; Acioglu et al., 2021). Additional evidence also points to dysfunctional endo-lysosomal machinery, as well as impaired calcium signaling and neuron-glia communication in AD (Canning et al., 1993; Söllvander et al., 2016; Mizuno et al., 2018; Verkhratsky, 2019; Habib et al., 2020). This general loss of astrocytic functionality seems to stem from early calcium-related events and impaired neuronal activity, which translates to reactive astrogliosis and synaptic dysfunction mediated by impaired glutamate, water, and ionic homeostasis, as well as faulty lipid metabolism in later stages of disease [for a detailed review, see (Brandebura et al., 2023)]. In addition, accumulation of pathologic tau has been observed in astrocytes in several disorders, including PSP, corticobasal degeneration, Pick’s disease, and aging-related tau astrogliopathy, leading to dystrophy and death of astrocytes (Leyns and Holtzman, 2017; Verkhratsky et al., 2023).

Genomic and transcriptomic studies also implicate several mutations and risk loci that impair normal astrocytic function, as well as the existence of brain region-specific reactive sub-states of astrocytes (Brandebura et al., 2023; Patani et al., 2023). For example, *APOE*, the strongest genetic risk factor for late-onset Alzheimer’s disease, is largely produced by astrocytes and microglia in the brain (Fagan et al., 1999). *APOE* imparts an allele-specific risk (*ε4* > *ε3* > *ε2*), with studies on *ε4* variants in iPSC-derived astrocytes showing cholesterol and matrisome pathway dysfunctions, poor neurotrophic function, and widespread transcriptomic and metabolic changes (Zhao et al., 2017; Lin et al., 2018; Tcw et al., 2022). Other genetic mutations, including those within *HTT* in HD, *LRRK2* in PD, and *Sod1* in ALS, have also been shown to impair normal astrocytic function and cause neuronal dysfunction, exacerbating neurodegeneration (Brandebura et al., 2023). Several single-cell and bulk transcriptomic studies have identified the existence of reactive states in disease-associated astrocytes, which exhibit a pro-inflammatory signaling environment that results in a loss of homeostatic astrocyte function and neuronal support (reviewed in detail in Brandebura et al., 2023; Patani et al., 2023). These reactive states do not represent a single binary state, but rather a spectrum of context-dependent phenotypes that vary by disease, brain region, disease stage, and interaction with other brain cell types. Given the substantial omics and functional data that already showcase the significance of glial cell types in neurodegenerative disease progression, it is increasingly important to identify regulatory drivers, allowing us to better understand how aging and other environmental factors lead to a disease phenotype. Mapping the DNA methylome will likely offer insights into these aspects and identify potential mechanisms that can better inform the therapeutic space. The next section will focus on evidence from DNA methylation studies indicating a role for glial cell types in neurodegenerative disease.

### Role for DNA methylation in astrocyte dysfunction in neurodegenerative diseases

DNA methylation has long been associated with pathological brain states. Several epigenome-wide association studies identify associations between differentially methylated CpG sites and the occurrence of neurodegenerative disease, as well as various stages of disease etiology. For example, in the case of PD, a recent EWAS of peripheral immune cells identified cell-type-specific differentially methylated regions (DMRs) associated with early stages of disease (Andersen et al., 2023), and a brain-region-specific EWAS identified regional alterations in DNA methylation associated with depressive symptoms in PD, enriched for neuronal subtype-specific transcriptomic changes in the substantia nigra (Harvey et al., 2025). Similarly, studies have observed DNA methylation-associated changes with HD, specifically showing epigenetic age acceleration in at least the frontal lobe, parietal lobe and cingulate cortex (Horvath et al., 2016). Other studies show differential methylation associated with age of disease onset in the cortex (De Souza et al., 2016), significant tissue-specific DNA methylation at 38 CpG sites in the *HTT* gene region (De Souza et al., 2016), and significant association of HD mutation status with DMRs at 33 sites in human blood (Lu et al., 2020). Whole blood methylome wide association studies in ALS have identified several CpG sites linked to disease status (Nabais et al., 2020; Hop et al., 2022), including those within genes enriched for metabolism and immune-related processes (Hop et al., 2022). Similar epigenetic age acceleration and altered DNA methylation profiles have also been identified in frontotemporal lobe degeneration (encompassing FTD) through EWAS studies in the brain and blood (Fodder et al., 2023; Murthy et al., 2023a). Finally, a growing body of EWAS studies have identified consistent methylation signatures associated with AD. DMRs within genes, including *ANK1, BIN1, ABCA7*, and *RHBDF2*, are associated with AD pathological burden in the cortex (De Jager et al., 2014; Lunnon et al., 2014). These modifications show region-specific associations with tau pathological burden, replicated across several cohorts. Additional significant CpG loci associated with AD include those within the *HOXA* gene cluster (Smith et al., 2018, 2021; Shireby et al., 2022). Beyond site-specific changes, accelerated epigenetic aging has also been observed in the prefrontal cortex of post-mortem AD brains, linking biological aging to disease pathology and cognitive decline (Levine et al., 2015). Other studies indicate some common methylomic patterns across different neurodegenerative disorders (Sanchez-Mut et al., 2016). Critically, while a large proportion of studies have observed overall tissue-specific or region-specific DMRs associated with neurodegenerative disease, cell-specific contributions are largely understudied.

When profiling DNA methylation from neuronal (NeuN+), oligodendrocytic (SOX10+) and mixed cell (NeuN-/SOX10-) fractions, Shireby et al. (2022) found that most differentially methylated sites reflect differences in the non-neuronal and non-oligodendrocyte fractions. Gasparoni and colleagues also identified neuronal and non-neuronal cell-type-specific DMRs associated with Braak stage, including enrichment of genes such as *ANK1* in the non-neuronal population (Gasparoni et al., 2018). Hypomethylation of CpG sites within the *APOE* gene is also implicated in non-neuronal cell types in AD brain (Tulloch et al., 2018). Similar changes are evident in *in vitro* studies. For instance, in generating “aged” neurons by direct lineage conversion, Mertens et al. (2021) show that iNs from individuals with AD do not show accelerated epigenetic aging. Since accelerated aging has been implicated in post-mortem AD brain tissue, it is possible that non-neuronal cell types contribute to this change. However, assessing all non-neuronal cells as a single fraction limits true cell-type resolution, given the diverse cell states of astrocytes and microglia, especially in disease conditions. Early studies show altered methylation at the promoters of glutamate transporter proteins, *GLT1* and *EAAT2*, in astrocytes of ALS patients (Yang et al., 2010). Moreover, distinct glial cell-type-specific functional changes have been observed in genetic studies using polygenic risk-score assessments for AD (Yang et al., 2023; Pratt et al., 2025). It would therefore be informative to assess astrocyte- and other glial cell-type-specific methylomes in neurodegenerative disorders to help unmask epigenetic shifts in pathological states may otherwise be obscured by cellular complexity in the adult brain. In this direction, our recent preprint using deconvolution-based meta-analysis identified AD-associated differential methylation at genes including *ADCY5* and *SLC5A5* in PFC astrocytes, as well as nominal significance for CpGs at *HOX* genes (Bhaskar et al., 2026). Other recent EWAS studies, including one non-peer-reviewed preprint, have begun characterizing microglia-specific methylomes in AD (Laroche et al., 2026; Zhu et al., 2026), highlighting the need for parallel advancements across cell types to fully map glial cell contributions to neurodegeneration.

Across neurodegenerative disorders, the strongest evidence for involvement of DNA methylation in disease pathology currently comes from EWAS and epigenetic aging studies (summarized in Table 1). Although these EWAS provide important evidence that DNA methylation is altered in neurodegenerative disease, most were performed in bulk brain tissue or in broad cell fractions. These designs are powerful for identifying disease-associated loci but do not, on their own, establish whether observed methylation changes arise within astrocytes. In the context of neurodegeneration, bulk methylation signals may represent neuronal loss, gliosis, vascular or immune-cell abundance, or shifts in astrocyte and microglial cell states. Therefore, astrocyte-specific interpretations should be cautiously made, unless supported by purified cell-specific or single-cell methylomic data. Critically, methylation changes observed in the diseased brain may also reflect shifts in abundance of astrocyte substates, stable epigenomic remodeling within a given astrocyte sub-population, or both. Therefore, integrating methylation data with single-cell transcriptomic and spatial approaches will be especially necessary in determining whether disease-associated methylation signatures correspond to specific astrocyte states.

**TABLE 1:** Age and neurodegenerative disease-associated brain DNA methylation changes in the context of astrocytes.

Context	Study design	Main methylation finding	Astrocyte-specific inference	References
Aging	Single cell multiomics approach	Increased DNA methylation with age at genes involved in synaptic function; more variation in excitatory versus inhibitory neurons.	Limited. Study did not investigate astrocyte-specific methylation.	Chien et al., 2024
	Bulk tissue meta-analysis	Increased methylation observed with age, which varies across brain regions.	Limited. Cell type proportions (neuron:glia) used as covariate, but cell-specific analysis was not performed.	Pellegrini et al., 2021
	Sorted (NeuN+/NeuN-) fractions in human cortex	Age-associated CpGs differ between neuronal and non-neuronal fractions, with limited overlap and sometimes opposing age trends.	Moderate. Non-neuronal fraction includes astrocytes, oligodendrocytes, microglia, OPCs, endothelial and other cells.	Gasparoni et al., 2018
	Deconvolution-based EWAS meta-analysis	Brain region-specific methylation differences observed with age; cell-type-resolved estimates suggest astrocyte-enriched hypermethylation at loci related to homeostatic response.	Moderate. Uses computational tools to generate astrocyte-specific methylome data; requires biological validation with sorted/single-cell datasets.	Bhaskar et al., 2026 (non-peer-reviewed preprint)
Parkinson’s Disease	Brain-region specific EWAS	Networks of DNA methylation associated with PD-related depression in the substantia nigra, localized to genes expressed in neuronal cells.	Limited. Cell type proportions (neuron:glia) used as covariate, but cell-specific analysis was not performed.	Harvey et al., 2025
Huntington’s Disease	Brain-region/cortex specific EWAS	Epigenetic age acceleration associated with HD; methylation associated with age of disease onset in cortex; cortical DNA methylation within the *HTT* gene associated with disease.	Limited. Cell proportions considered as covariates; no cell-specific analysis performed.	De Souza et al., 2016; Horvath et al., 2016
Frontotemporal dementia	Frontal cortex-specific EWAS meta-analysis	Identified two differentially methylated loci associated with FTD.	Limited. Cell proportions considered as covariates; no cell-specific analysis performed.	Fodder et al., 2023
Alzheimer’s disease	Bulk cortex EWAS and meta-analyses	Reproducible DMRs located within *ANK1, BIN1, ABCA7* etc., associated with AD pathology; differentially methylated sites within *HOX* gene clusters associated with AD; accelerated epigenetic aging associated with plaque status.	Limited. Cell proportions considered as covariates; no cell-specific analysis performed.	De Jager et al., 2014; Lunnon et al., 2014; Smith et al., 2018, 2021; Shireby et al., 2022
	Sorted cell fractions (NeuN+, SOX10+, NeuN/SOX10-)	Large proportion of differentially methylated sites associated with AD stem from NeuN/SOX10- fractions.	Moderate. NeuN/SOX10- fraction includes astrocytes, microglia, endothelial cells and other cells.	Shireby et al., 2022
	Sorted (NeuN+/NeuN-) fractions	Several DMRs identified within both fractions; DMR at *ANK1* enriched in non-neuronal fraction.	Moderate. Non-neuronal fraction includes astrocytes, oligodendrocytes, microglia, OPCs, endothelial and other cells.	Gasparoni et al., 2018
	Sorted (NeuN+/NeuN-) fractions; investigating DNA methylation within *APOE*	Hypomethylation at CpG sites within the *APOE* gene in NeuN- fraction.	Moderate. Non-neuronal fraction includes astrocytes, oligodendrocytes, microglia, OPCs, endothelial and other cells.	Tulloch et al., 2018
	Deconvolution-based EWAS meta-analysis	Differential methylation associated with AD is cell-type specific; prefrontal cortex astrocytes show differential methylation at sites within *ADCY5, SLC5A5*, and other genes.	Moderate. Uses computational tools to generate astrocyte-specific methylome data; requires biological validation with sorted/single-cell datasets.	Bhaskar et al., 2026 (non-peer-reviewed preprint)

## Concluding remarks and future perspectives

There is an ever-growing recognition of astrocytes as architects of brain health, and increased awareness of their contribution to the etiology and pathogenesis of various neurodegenerative disorders. Astrocyte lineage commitment, development, and functionality are deeply linked to the methylation status of glial-lineage genes. During early neurodevelopment, DNA methylation at genic promoters (e.g., *GFAP*) acts as a “lock” to ensure timely gliogenesis. It is not unfathomable that such regulatory blocks falter with age and with disease burden. Whether such age-associated epigenetic drifts are causal to the existence of reactive, pro-inflammatory astrocytic sub-states remains to be seen. A deeper understanding of the astrocyte methylome will allow the distinction of cellular states relevant to chronological aging, as well as neurodegeneration. It is also worth noting that the methylome interacts with other layers of the epigenome, including histone modifiers and non-coding RNAs, to regulate cellular function. While these were not a focus of this review, future studies should focus on integrating these diverse landscapes to collectively understand astrocytic response to aging and disease. Epigenome-wide association studies have identified key shifts in DNA methylation across several disorders, as well as with aging. The fact that these studies find significant pathological associations in non-neuronal cell types suggests the existence of aberrant DNA methylation in these cells. However, the use of either bulk tissue or non-discrete cell sorting (e.g., sorting only for NeuN+/NeuN- cells) masks cell-specific signals, and as such, profiling of cell-type-specific methylation patterns may provide a better understanding of disease pathogenesis. Recent advances in high-throughput cell sorting, single-cell omics methodologies, and cell-type deconvolution algorithms now offer an unprecedented opportunity to isolate cell-specific signatures of disease. The study of the astrocyte methylome is an exciting and highly promising avenue, which may provide a clearer picture of the primary drivers of aging and neurodegeneration, as well as elucidate pathogenic disease mechanisms that may lead to the identification of new targets for potential methylation-targeted therapeutics.
